# Immunogenic and antigenic analysis of recombinant NSP1 and NSP11 of PRRS virus

**DOI:** 10.1002/vms3.699

**Published:** 2022-01-12

**Authors:** María Josefina Contreras‐Luna, Gladis Fragoso‐Gonzalez, René Alvaro Segura‐Velázquez, Jacquelynne Brenda Cervantes‐Torres, Rogelio Alonso‐Morales, Luis Alfonso Ramírez‐Martínez, Dolores Adriana Ayón‐Núñez, Raúl José Bobes, José Ivan Sánchez‐ Betancourt

**Affiliations:** ^1^ Laboratorio de Investigación del Departamento de Medicina y Zootecnia de Cerdos Facultad de Medicina Veterinaria y Zootecnia Universidad Nacional Autónoma de México (UNAM) Mexico City Mexico; ^2^ Unidad de Investigación Facultad de Medicina Veterinaria y Zootecnia Universidad Nacional Autónoma de México (UNAM) Mexico City Mexico; ^3^ Instituto de Investigaciones Biomédicas Universidad Nacional Autónoma de México (UNAM) Mexico City Mexico; ^4^ Facultad de Medicina Veterinaria y Zootecnia Laboratorio de Genética Molecular Universidad Nacional Autónoma de México (UNAM) Mexico City Mexico; ^5^ Facultad de Medicina Veterinaria y Zootecnia Departamento de Microbiología e Inmunología Universidad Nacional Autónoma de México (UNAM) Mexico City Mexico

**Keywords:** betaarterivirus, neutralization, nonstructural proteins, PRRS

## Abstract

**Background:**

Porcine reproductive and respiratory syndrome virus (PRRSV) is an enveloped RNA virus in the order Nidovirales, family *Arteriviridae*, genus *Betaarterivirus*. Antibodies against nonstructural proteins (NSPs) from this virus can be found in pigs starting 4 days postinfection and they remain detectable for several months.

**Objective:**

The goal of this study was to evaluate the immunogenicity and antigenic properties of recombinant proteins NSP1 and NSP11 expressed in *Escherichia coli* cells, as well as to assess the neutralization activity that they elicit.

**Methods:**

We obtained the complete *ORF‐1* genes coding for NSP1 and NSP11 from PRRSV using the VR‐2332 strain. Cloning was performed with the pET23a(+) vector with a histidine tag (His6), linearized by restriction enzyme digestion; the expression of the NSP1 and NSP11 clones was induced in OverExpress C41(DE3) chemically competent cells. Recombinant proteins were used to generate hyperimmune sera and we perform serological assays to confirm neutralizing antibodies.

**Results:**

The expressed recombinant NSP1 and NSP11 were found to be immunogenic when injected in pigs, as well as demonstrated higher specificity in recognition of antigen in field sera from pigs positive infected with PRRSV. Furthermore, both NSP1 and NSP11 recombinant proteins elicited PRRSV neutralizing antibodies.

**Conclusions:**

In this study, we demonstrated the immune humoral response to NSP 1 and NSP11, and neutralizing‐antibody response to PRRSV VR2332 strain in sera from hyperimmunized pigs.

## Graphical abstract

Immunogenicity and antigenic properties of recombinant proteins NSP1 and NSP11. NSP1 and NSP11 recombinant proteins elicited PRRSV neutralizing antibodies. Control of PRRSv using recombinant NSPs.

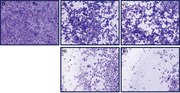

## 1 INTRODUCTION

The causal agent of porcine reproductive and respiratory syndrome (PRRS) is an enveloped RNA virus from the order Nidovirales, family *Arteriviridae*, genus *Betaarterivirus*, subgenus *Ampobarterivirus*. The PRRS virus (PRRSV) has two known variants: *Betaarterivirus suid 1*, previously known as PRRSV‐1, and B. *suid 2*, previously known as PRRSV‐2 (King et al., 2018). Because PRRS increases fetal death rates by 20%–30% (Goyal, 1993), it has a strong negative economic impact on pork production. In the United States, economic losses due to PRSS have been estimated at 664 million dollars annually. On pig farms alone, losses are calculated at 302.06 million dollars, chiefly due to lower prices at time of weaning (Holtkamp et al., 2013; Neumann et al., 2005).

There are commercial vaccines available on the market worldwide, the most widely used contain the entire genome based on modified active virus (MLV) or inactive form. Most PRRS vaccines elicit specific humoral and cellular immune responses that confer protection against homologous parental strains and partial protection to heterologous strains, but there are safety concerns, such as a high mutation rate that causes reversion to virulence and recombination between vaccines and field strains ( Renukaradhya et al., 2015), in addition, the response they induce is late, which confers partial protection when the animals face the field virus. (Renukaradhya et al., 2015).

While previous studies have focused on the immune response against PRRSV structural proteins like N or GP5, their high variability and poor individual response to the virus have prevented us from finding a feasible strategy against this pathogen agent (Chand et al., 2012; Renukaradhya et al., 2015). Besides, we have demonstrated the neutralization capacity of NSP1 (Leng et al., 2017; Su et al., 2019), which have been used in this study.

PRRSV infects cells that participate in both the innate and the adaptive immune response, such as macrophages ‘particularly with alveolar location and dendritic cells’ (Lunney et al., 2010; Rossow, 1998; Welch & Calvert, 2010). NSP 11 has a function similar to NSP1 and antagonizes IFN type I, specifically IFNβ production (Montaner‐Tarbes et al., 2019). An anti‐NSP11 monoclonal antibody (mAb) created to test reactivity against genotype 1 and genotype 2 PRRSV was able to induce humoral immune response in PRRSV infected pigs. Furthermore, a B‐cell epitope on the surface of NSP11 using a specific mAb was also able to induce humoral immune response in pigs infected with PRRSV (Jiang et al., 2017). The goal of this study was to evaluate immunogenicity, as well as the neutralization ability of recombinant NSP1 and NSP11 to contribute towards potential development of new generation PRRSV vaccines.

## 2 MATERIALS AND METHODS

### 2.1 Propagating PRRSV

To replicate PRRSV, we infected MA‐104 line cells (ATCC® CRL‐2378.1; Benfield et al., 1992) with the PRRSV ATCC® VR‐2332 strain. The cells were propagated in Eagle's minimal essential medium (MEM), supplemented with L‐glutamine and 10% fetal bovine serum (FBS). We resuspended 5 × 10^6^ cells in 150 μl, adding as much of Dulbecco's modified MEM (DMEM) supplemented with 1000 UI penicillin/streptomycin and 2% FBS as required to complete 5 ml. Virus replication was verified by quantitative RT‐PCR (qRT‐PCR) using the POCKIT™ PRRSV Reagent Set kit, following the manufacturer's directions, in a Rotor‐Gene thermocycler (Corbett Research, Sydney, Australia).

### 2.2 Producing recombinant proteins, cloning and expressing NSP1 and NSP11

We designed specific primers to amplify the entire *ORF‐1* genes that code for NSP1 and NSP11 from the PRRSV ATCC VR‐2332 strain, via RT‐PCR. These primers end sequences incorporated BamHI, XhoI and EcoR1 (Table 1), which correspond to enzymatic restriction sites. We obtained viral RNA using the OneStep RT‐PCR Kit® (Qiagen, Hilden, Germany), following the manufacturer's instructions. The samples were amplified for 35 cycles, preceded by an initial cDNA cycle at 50°C for 30 min and a denaturing stage at 95°C for 15 min. A final extension cycle was performed at 72°C for 10 min. We used a mastercycler gradient thermocycler (Eppendorf, Hamburg, Germany) to carry out the RT‐PCR, and both primers and test conditions are shown in Table 1.

**TABLE 1:** Primers and reaction conditions to generate ORF‐1 NSPs from the PRRSV genome

NSP	Sequence (5′‐ 3′)	Size (pb)	Temperature (°C)	Cycle time (s)
**NSP1** ^a^	**F**:5′GCGGATCC/GAATTC/TCTGGGATACTTGATCGGTG EcoR1 **R**:5′GGTGGTGGTG/CTCGAG/GCCGTACCACTTGTGAC Xho1	1148	A 94 B 57 C 72	60 60 120
**NSP11**	**F**:5′AATGGGTCGC/GGATCC/AGCGTGTTGTAGATTCTCTCCG BamHI **R**:5′GGTGGTGGTG/CTCGAG/CATAGCTGGCAAGCTGATACC XhoI	750	A 94 B 54 C 72	60 60 120

PCR denaturation (A), alignment (B) and extension (C) temperatures. In each stage, the temperature was maintained for the time shown in the next column.

^a^ Modified from Brown et al. (2009).

We verified the presence of amplified fragments on 2% agarose gels with tris‐acetate‐EDTA buffer (TAE 1X) by staining with ethidium bromide and observing samples in a transilluminator. We used a pET23a(+) vector with a histidine tag (His6), linearized by restriction enzyme digestion, for cloning with an In‐Fusion HD Plus Complete Cloning System kit (Clontech Laboratories, Mountain View, CA), following the manufacturer's instructions. BL21 Stellar Competent Cells (Clontech Laboratories) were transformed with the recombinant plasmid. To verify cloning (vector‐insert), the cultures were purified with the QIAprep® Spin Miniprep kit (Qiagen). The identity of obtained amplicons was verified by sequencing. The expression of the NSP1 and NSP11 clones was induced in OverExpress™ C41(DE3) chemically competent cells (Sigma‐Aldrich, St. Louis, MO) with 1 mM IPTG 1 mM at 37°C for 6 and 8 h, respectively. Induced cells were recovered and lysed in a buffer containing Tris‐HCl 50 mM pH 8.0, NaCl 100 mM and EDTA 1 mM. The expressed proteins were assessed by 12% SDS‐PAGE at 110 V for 140 min and stained with Coomassie blue.

### 2.3 Purifying recombinant NSP1 and NSP11

Histidine‐tagged, recombinant NSP1 and NSP11 were purified in a HisTrap HP affinity column (GE Healthcare Life Sciences, Chicago, IL) by affinity chromatography with an ÄKTAprime Plus system (GE Healthcare Life Sciences), following the manufacturer's protocol. The Lowry method was chosen to quantify eluted proteins.

### 2.4 Characterizing NSP1 and NSP11 by western blot

We transferred eluted proteins to a PVDF membrane and performed 1D/2D SDS‐PAGE at 110 V for 1 h (4°C). Following protein separation, the membranes were blocked with 3% BSA in PBS‐T for 1 h. After this, membranes were washed with PBS‐T and incubated for 1 h with a mouse monoclonal antibody (Roche Diagnostics, Basel, Switzerland) targeting the His6 label of the recombinant product, in a 1:4000 dilution. The membranes were then washed with PBS‐T and incubated for 1 h with mouse anti‐mouse polyclonal antibody, IgG isotype, linked to horseradish peroxidase (Sigma) in a 1:2000 dilution. We visualized the reaction using 3 mg/ml 3,3‐diaminobenzidine (Sigma) in PBS‐T and 30% hydrogen peroxide in a 1:1000 dilution.

### 2.5 Producing hyperimmune sera to NSPs

We immunized four York–Landrace pigs from a PRRS‐free farm, two pigs each received either recombinant NSP1 or NSP11. The absence of anti‐PRRSV antibodies was verified using the commercial HerdChek PRRS 2XR Ab ELISA test (IDEXX, Westbrook, MA). Each pig was vaccinated four times at 15 day intervals with 100 μg protein doses in a 1:1 protein‐Montanide adjuvant emulsion, via deep intramuscular route in the neck. We established a baseline using a blood sample taken before the first immunization. Then, blood samples were taken 7 days following each immunization, to monitor serum antibody titres and determine when peak antibody titres occurred via ELISA tests.

### 2.6 Western blot for hyperimmune sera

To confirm PRRSV recognition by pig hyperimmune sera, we performed a western blot assay. MA‐104 cells were infected with the PRRSV ATCC VR‐2332 strain, then 48 h after infection we recovered virions from the infected cell monolayer with the RIPA lysis and extraction buffer (Thermo Scientific, Waltham, MA), following the manufacturer's instructions. Viral particles were transferred to a PVDF membrane and performed a 1D/2D SDS‐PAGE at 110 V for 1 h (4°C). Upon separation, the membranes were blocked with 3% BSA in PBS‐T for 1.5 h, they were then washed with PBS‐T and incubated for 2 h with hyperimmune NSP1 or NSP11 serum in 1:25 and 1:50 dilutions, respectively. We washed the membranes three times with PBS‐T and incubated for 1 h with anti‐swine polyclonal antibody, IgG isotype, linked to horseradish peroxidase (Sigma) in a 1:2000 dilution. The reaction was visualized using 3 mg/ml 3,3‐diaminobenzidine (Sigma) in PBS‐T and 30% hydrogen peroxide in a 1:1000 dilution.

### 2.7 Antigenicity of recombinant NSPs

To determine the antigenicity of the recombinant proteins, we developed an ELISA method (ELISA “in house”) using purified recombinant NSPs as antigen sources and following the previous report (García‐Plata, 2016). We used 50 negative and 50 positive sera (commercial kit) from pigs belonging to farms where PRRSV is present, and then compared the results with an ELISA “in house”.

Before tests on field sera were run, we tested the purified recombinant proteins against known positive sera identified with high antibody titres and found recognition by these sera against both proteins.

We coated 96‐well plates (MicroWell, Nunc‐Immuno, Thermo Scientific) with the purified NSP1 or NSP11 recombinant protein using 0.1 μg/ml per well and diluted in carbonate buffer. The wells were incubated overnight at 4°C, then washed three times with 3% PBS‐Tween BSA, blocked with 1% PBS‐BSA and incubated for 60 min at 37°C, then the plates were washed three times with PBS‐Tween.

Serum samples were diluted 1:100 in 1% PBS‐BSA dilution buffer on each plate, with hyperimmune sera for each recombinant NSP included as positive controls and baseline sera from the hyperimmunized pigs included as negative controls. Plates were incubated for 60 min at 37°C,then washed three times with PBS‐Tween 3%. We added 100 μl of 1:120 000 dilution peroxidase‐linked secondary antibody (anti‐pig IgG, Sigma‐Aldrich) to each well and then incubated the plates for 60 min at 37°C. Each serum was tested in duplicate. After washing the plates as described above, the reaction was visualized using 100 μl per well with tetra‐methyl benzidine (TMB, Invitrogen, Carlsbad, CA) and stopped after 30 min with 0.2 M sulfuric acid. Absorbance was measured in an Epoch monochromator spectrophotometer (BioTek, Winooski, VT) at a wavelength of 450 nm.

The cut‐off point to distinguish negative and positive samples was calculated as the mean reading of negative controls plus twice the standard deviation. Values below this limit were considered negative and any value above this point was regarded as positive. We calculated assay sensitivity as *TP*/(*TP* + *FN*) × 100, and specificity as *TN*/(*TN* + *FP*) × 100, where *TP, TN, FP*, and *FN* stand for the number of true negative, true positive, false positive and false negative results, respectively (OIE, 2019). We also calculated the kappa concordance coefficient.

### 2.8 Serum neutralization

A serum neutralization assay was performed to estimate the NSPs neutralizing capacity of hyperimmune sera. Sera were heat inactivated at 56°C for 30 min, and then serum neutralization assay was performed following the method described by Leng et al., 2017 with some modifications. We diluted hyperimmune sera for NSP1 or NSP11 using a two‐fold serial dilution in MEM. Then, 100 μl of each diluted sample was mixed with an equal volume of the PRRSV (ATCC, VR2332) strain (100, 300, 500 and 1000 TCID50%).The mixtures were incubated for 1 h at 37°C and then transferred to a 96‐well plate containing confluent MA‐104 cell monolayers prepared 24 earlier. The plates were incubated at room temperature for 60 min, and then they were kept at 37°C under 5% CO_2_ for 96 h and monitored daily for CPE. The presence of virus‐specific CPE in each well was recorded after 96 h of incubation. The neutralization antibody (NA) titre of each hyperimmune serum sample against the PRRSV was calculated using the Reed‐Müench method.

## 3 RESULTS

### 3.1 Replication and cloning of recombinant proteins

**FIGURE 1:**
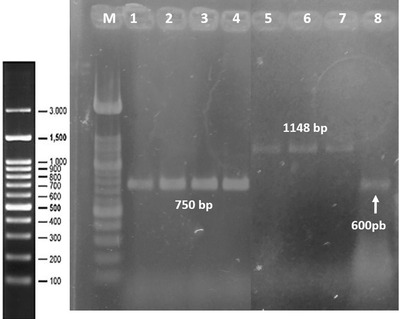
RT‐PCR Gradients for amplification of the Nsp1 and Nsp11 proteins. 2% agarose gel. Amplicons used by RT‐PCR were analyzed in an electrophoresis chamber using a molecular weight marker of 100–12,000 bp and 100–1000 bp (M). Nsp1 (1–4), Nsp11 (5–7), 58° (8) Positive control (ORF7)

**FIGURE 2:**
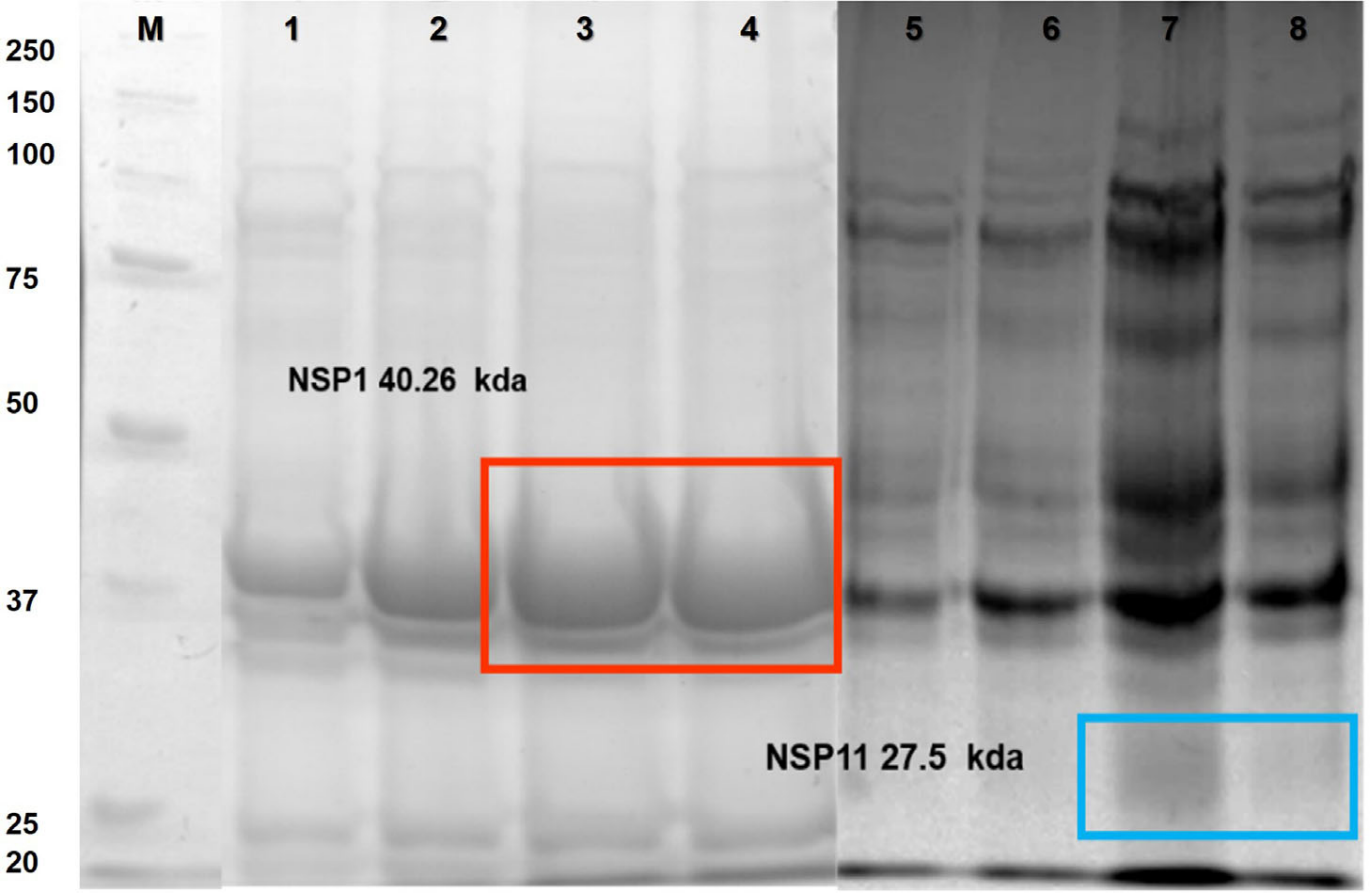
NSP1 expression in OverExpress™ chemically competent cells. 12% polyacrylamide gel. Total NSP1 proteins (1–4) and NSP11 (5–8). All samples were analyzed using a molecular weight marker as a reference value in kDa (M). Time: NSP1 = 2 h (2), NSP11 = 4 h (3), NSP1 = 6 h (4), NSP11 = 0 h (5), NSP11 = 2 h (6), NSP11 = 4 h (7), NSP11 = 6 h (8)

We confirmed replication of PRRSV in MA‐104 cells via qRT‐PCR (Table 2). The ORF‐1 genes amplified were of 1148 bp is for NSP1 and 750 bp for NSP11 (Figure 1). NSP1 and NSP11 recombinant were analyzed by SDS‐PAGE (Figure 2). Un‐purified NSP1 and NSP11 are shown in lanes 1 and 2, respectively (Figure 2). Purified NSP1, with an expected molecular weight of 40.26 kDa, is shown in lanes 3 and 4, while NSP11, with an expected molecular weight of 27.5 kDa (including N’‐terminal poly‐histidine), is shown in lanes 7 and 8.

**TABLE 2:** PRRSV concentration as determined by qRT‐PCR in different cell passages

Name	Type	CT	Copies/ul
PRRS C(+)	Test positive control	29.61	199 679 036
VR2332	Virus 1st passage MA‐104	32.71	1 462 606
VR2332	Virus 2nd passage MA‐104	32.65	1 619 783
VR2332	Virus 3rd passage MA‐104	29.86	133 309 614

### 3.2 Purification and yield of recombinant proteins

**FIGURE 3:**
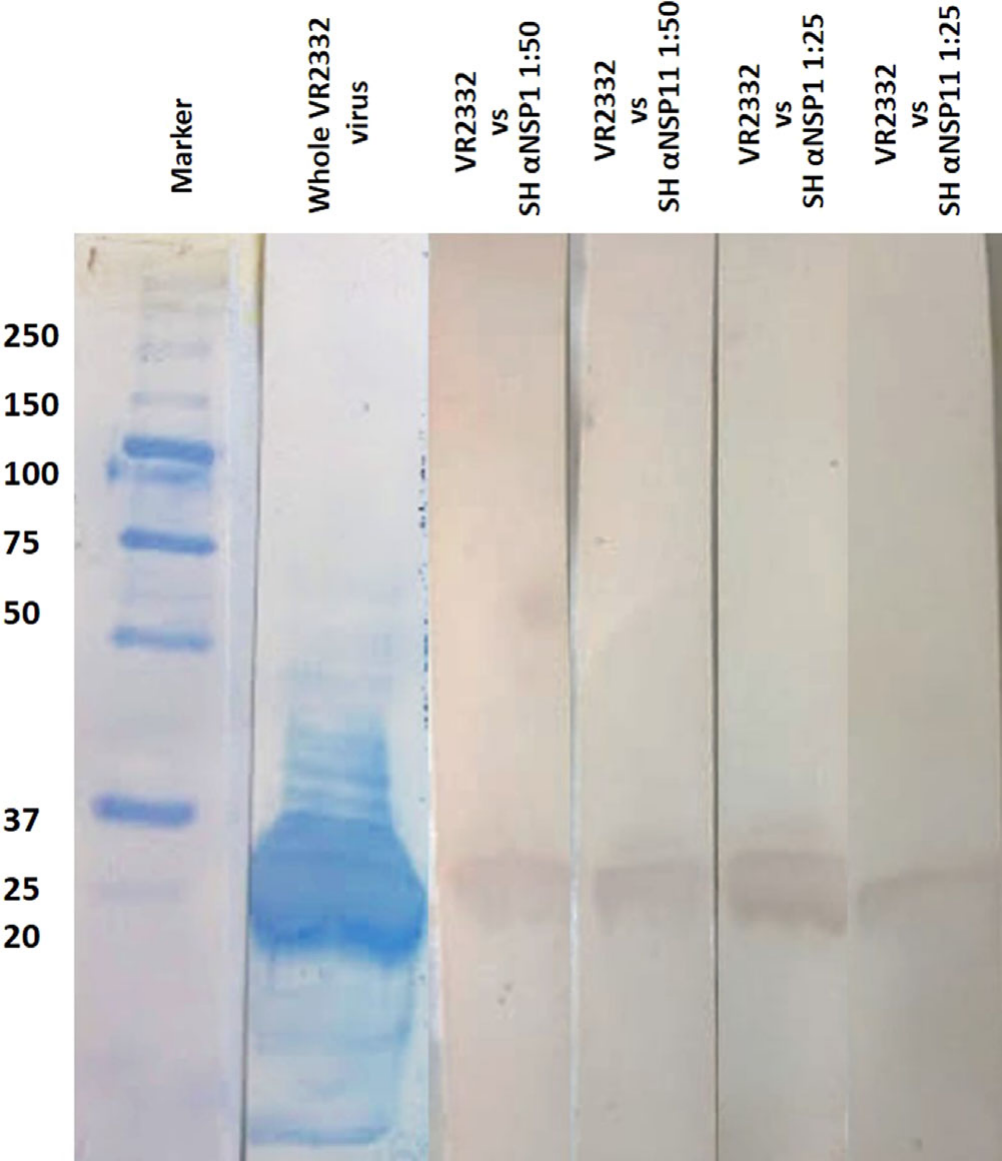
Hyperimmune serum against NSP1 and NSP11 detected by Western blot with anti‐pig IgG, compared with whole PRRSV VR2332 strain. Molecular weight marker as a reference value in kDa (M); hyperimmune serum for NSP1 (SH αNSP1); hyperimmune serum for NSP11 (SH αNSP11)

Expressed proteins are located in the insoluble fraction of bacterial cells and were purified by inclusion antibodies under denaturation conditions. We obtained fractions in the concentration peaks in a polyacrylamide gel, and the presence of pure proteins was verified. Production yield was 597.5 μg/ml for NSP1 and 201.7 μg/ml for NSP11. Both recombinant proteins reacted with anti‐His6 antibodies in immuno transfer. We found antigen and complete virus recognition for NSP1 or NSP11 in the different dilutions that were used in the western blot testing of hyperimmune sera (Figure 3).

### 3.3 Hyperimmune sera

**FIGURE 4:**
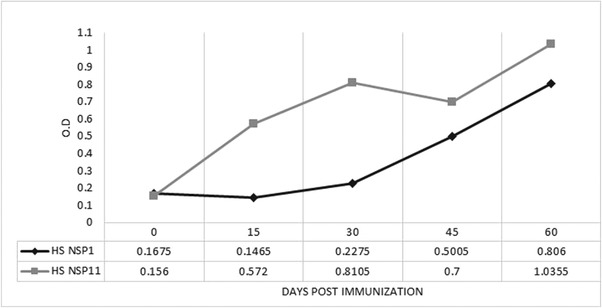
Time‐course for seroconversion of pigs inoculated with recombinant NSP1 and NSP11. Serum samples were taken on days 0, 15, 30, 45 and 60 postimmunizations

Pigs immunized with recombinant NSPs seroconverted at different times postinoculation (Figure 4). Specific antibodies against NSP1 were detectable between days 30 and 45 postinfection and anti‐NSP11 antibodies were detected on day 15 postinfection, the highest levels were observed on day 60, with an optical density value of 1.035.

### 3.4 Specificity and sensitivity of recombinant proteins

Sera obtained from five different states of Mexico were tested with commercial ELISA kit. We used 50 positive sera and 50 negative sera to confront with NSP1 and NSP11 in the ELISA in house. The recombinant NSP1 detected specific anti‐NSP1 antibodies in 92 out of 100 serum samples (98.03% specificity and 53.8% sensitivity), while recombinant NSP11 detected 99 out of 100 positive serum samples (99% specificity and 50.5% sensitivity) (Table 3).

**TABLE 3:** Comparison between ELISA, ELISA NSP1 and ELISA NSP11

STATE	ELISA		ELISANSP1		ELISANSP11		Reactivity (positive/negative)		
	(+)	(−)	(+)	(−)	(+)	(−)	ELISA	Nsp1	Nsp11
***Sonora***	10	10	19	1	20	0	10/10	19/1	20/0
***Jalisco***	10	10	15	5	20	0	10/10	15/5	20/0
***Michoacán***	10	10	20	0	20	0	10/10	20/0	20/0
***Guanajuato***	10	10	20	0	20	0	10/10	20/0	20/0
***Yucatán***	10	10	18	2	19	1	10/10	18/2	19/1
TOTAL	**50**	**50**	**92**	**8**	**99**	**1**	**50/50**	**92/8**	**99/1**
***%***	50%	50%	92%	8%	99%	1%			

### 3.5 Neutralization antibody

**FIGURE 5:**
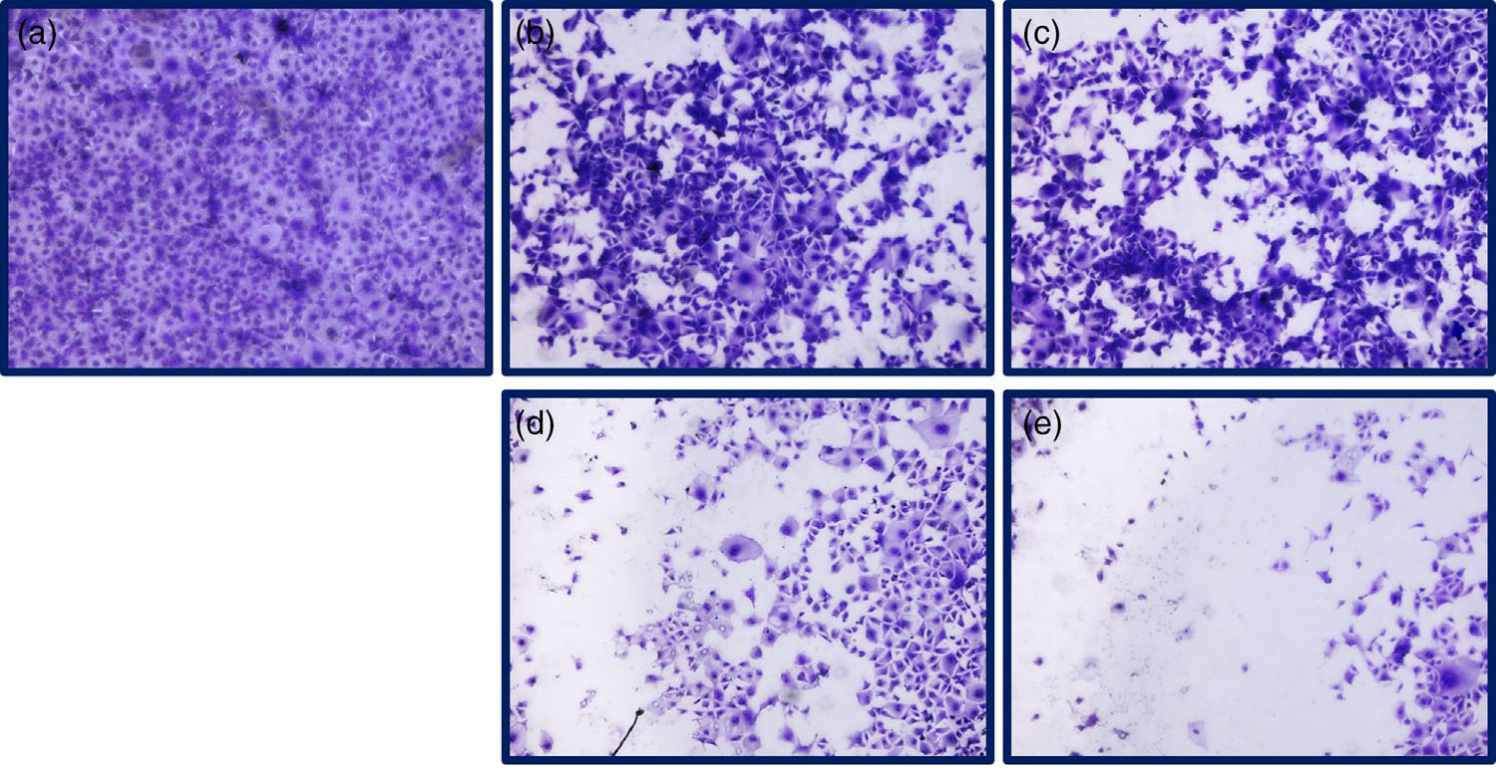
Cytopathic effect of PRRSV VR2332 strain at different concentrations. (a) Negative control; (b) 100 ID; (c), 300 ID; (d) 500 ID; and (e) 1000 ID. MA‐104 cell monolayer, confluence 80% at 96 h postinfection. Microphotography under inverted microscope 40× and crystal violet stain. ID: infective dose

We detected antibodies for NSP1, NSP11 and PRRSV in hyperimmune sera. Negative serum samples showed no response to the test and the cytopathic effect to differentiate positive from negative responses was evident in cell cultures (Figure 5). Hyperimmune sera showed a better response at lower infecting doses (100 and 300). For NSP1, we obtained protective titres of 1:32 at an infectious dose of 100, and of 1:16 titres at an infectious dose of 300. We found protective titres of 1:8 and 1:4 for NSP11 at infectious doses of 100 and 300, respectively. At higher infective doses (500 and 1000) we observed lower serum neutralizing responses (Table 4).

**TABLE 4:** Neutralizing antibody titres for different PRRSV infecting doses in a serum neutralization assay

**Serum**	**Titre with infecting dose**			
	**100**	**300**	**500**	**1000**
SH NSP1	32	16	8	4
SH NSP11	8	4	4	4
SH VR2332	8	4	4	2
Negative serum	0	0	0	0

Titres are expressed as the inverse of serum dilution.

## 4 DISCUSSION

No effective anti‐PRRSV vaccine has been developed yet, and although DNA, subunit and attenuated vaccines have been tested, their potential as a substitute of the MLV anti‐PRRS vaccine currently in use, is uncertain (Nan et al., 2017). An early and long‐lasting immune response against PRRSV NSPs could be the key to this goal.

Some studies have reported the production of hyperimmune sera against PRRSV in mice (Bi et al., 2017), or by inoculating pigs with peptides (Díaz et al., 2009). In both these cases, the hyperimmune serum is targeted to B‐cell epitopes, which have a higher probability of generating antibodies. On the other hand, PRRSV NSPs play a key role in the processing and maturation of the virion structural repertoire. Swine are known to mount antibody responses to these proteins with significant cross‐reactivity, which seems to be equal to or higher than that induced by nucleocapsid protein (Johnson et al., 2007).

Additionally, early antibody detection could play an important role in surveillance and in the implementation of preventive measures against the virus PRRSV inhibits the expression of type‐I (α/β) interferon (IFN), which in turn induces the synthesis inhibition of several antiviral proteins (Darwich et al., 2010; Yoo et al., 2010). Type‐I IFN expression interference is largely due to the capacity of NSPs to inhibit the IFN‐β promoter by blocking IRF3 translocation and inhibiting NF‐κB (Rascón‐Castelo et al., 2015). Additionally, the expression of IL‐10 is induced, while that of IL‐1 and TNF‐α is inhibited. This causes a limited inflammatory response, which in turn leads to a less effective adaptive response, resulting in delayed production of neutralizing antibodies until 3–4 weeks postinfection (Flores‐Mendoza & Hernández, 2010).

The Western blot assay results (Figure 3) also indicated that whole PRRSV cultured in MA‐10^4^ cells can be recognized by the hyperimmune sera specific for whole NSPs. This is very important because NSPs are available for antigenic presentation through MHC‐I and ‐II from the earliest moments of infection. Cytolytic infection also releases viral proteins into interstitial spaces, generating a strong antibody response, equivalent to the immune response to structural proteins (Brown et al., 2009). Early response to NSPs indicates that these proteins are shown on the surface of infected cells for their presentation to B cells. On the other hand, anti‐NSP antibodies can be detected early during viral infection at levels that depend on their immunogenicity and abundance (Johnson et al., 2007). Neutralizing antibody titres are produced in piglets inoculated with chimeric viruses, where the 5′ untranslated region (UTR) + open reading frame (ORF) 1a, ORF1b and ORF2‐7 + 3′UTR was exchanged between two infectious strains, that study also found that ORF1a is also a PRRSV neutralization region (Leng et al., 2017); several works have focused in detecting antibodies against viral structural proteins (Cancel‐Tirado et al., 2004; Díaz et al., 2009; Wootton et al., 1998). These justifies the use of NSPs as components of a candidate vaccine that could elicit an early, protective immune response against PRRSV.

The time‐course of antibody response showed that antibody response to NSP7 is comparable to the response to NSP1 and NSP2, as well as to the antigens used in the IDEXX commercial ELISA kit (Brown et al., 2009). Recombinant NSP1 and NSP11 demonstrated to be immunoreactive: NSP1 was recognized by 98.03% of serum samples known to be positive, and NSP11 was recognized by 100% of those samples.

Our study found that several serum samples previously labelled as negative are now identified as positive, due to higher test sensitivity when using NSP1 or NSP11 as the antigen source. Given the current lack of an effective vaccine, there exists a great need for the development of a high‐sensitivity plate test that uses antigens like the NSPs used in this study. These antigens are expressed early on during the infectious process and are recognized by the immune system before the structural proteins are. Additionally, this response lasts for several months. Johnson et al. (2007) found that NSPs are available for antigenic presentation through MHC‐I and ‐II at the earliest moments of infection. Because cytolytic infection also releases viral proteins into interstitial spaces, we can know that an induced strong antibody response to NSPs will be comparable to the immune response elicited by structural proteins. Furthermore, sustained anti‐NSP antibody titres have been found for up to 202 days, while the antibody response to the N protein gradually decreases after 126 days (Brown et al., 2009).

Neutralizing titres in hyperimmune sera against NSP1 and NSP11 indicate that the antigenic activity could be relevant in the individual response. Furthermore, that at some point during infection, they may play an important role in the neutralizing activity of antibodies against the virus. Various studies have reported PRRSV antibody neutralization, mainly using hyperimmune sera against proteins GP5, M or N (Gonin et al., 1999; Loemba et al., 1996; Yoon et al., 1994). However, few reports show that there is a neutralization region in PRRSV ORF1, mainly in NSP2, which has been better studied so far (Leng et al., 2017).

The main neutralizing activity against PRRSV has been reported to target the GP5 protein (Lopez & Osorio, 2004; Popescu et al., 2017; Vashisht et al., 2008). However, our results suggest that NSPs, in this case NSP1 and NSP11, can be recognized by the immune system of hosts that can then elicit an antibody response with neutralizing capacity and NSPs are the first viral proteins to be synthesized in cells infected by PRRSV. These suggests that both proteins

contain highly antigenic regions, and are well conserved across different PRRSV strains. Thus NSP‐based immunogens could induce protection against different viral strains, with an effective and rapid response to the infection, preventing the immunomodulatory effects of the virus (Rascón‐Castelo et al., 2015).

Recombinant protein‐based approaches allow us to rapidly and easily synthesize large amounts of a product whose characteristics are very similar to those of a natural protein, and as epitope‐based vaccines have several benefits including safety, high specificity and ease of use (Nan et al., 2017).  Herein, we demonstrated that NSP1 and NSP11 open a new approach to produce a protective recombinant vaccine to control PRRS.

These suggests that both proteins contain highly antigenic regions, however further studies are needed to confirm whether this humoral response provide cross‐neutralization against heterologous PRRSV strains.

## 5 CONCLUSIONS

Our results indicate that recombinant PRRSV NSPs can be antigenic and immunogenic. This study is an initial exploration to propose the use of recombinant proteins as a novel immunogenic approach against PRRSV and/or to develop a more precise diagnostic tool.

## CONFLICT OF INTERESTS

Authors declare no conflict of interests.

## AUTHOR CONTRIBUTIONS

Contreras‐Luna: investigation; writing – original draft; writing – review and editing; formal analysis. Fragoso‐Gonzalez: writing – review and editing. Segura‐Velázquez; resources. Cervantes‐Torres: investigation. Alonso Morales: writing – review and editing. Ramírez‐Martínez: investigation; writing – review and editing; formal analysis. Ayón‐Núñez: methodology; validation. Bobes: methodology; validation; resources. Sánchez‐Betancourt: conceptualization; resources; supervision; writing – review and editing.

## ETHICAL STATEMENT

The Committee for Animal Experiments at the Faculty of Veterinary Medicine and Animal Husbandry, FMVZ‐UNAM (SICUAE, number DC‐2018/2‐2) granted ethical approval for the use of experimental animals.

### PEER REVIEW

The peer review history for this article is available at https://publons.com/publon/10.1002/vms3.699

## Data Availability

The data related of this experimental study are available on request from the corresponding author.

## References

[vms3699-bib-0001] OIE . (2019). Principles and methods of validation of diagnostic assays for infectious diseases. In Manual of diagnostic tests and vaccines for terrestrial animals 2019. OIE. (World Organisation for Animal Health), pp. 1–18. https://www.oie.int/fileadmin/Home/eng/Health_standards/aahm/current/chapitre_validation_diagnostics_assays.pdf

[vms3699-bib-0035] Benfield, D. A. , Nelson, E. , Collins, J. E. , Harris, L. , Goyal, S. M. , Robison, D. , Christianson, W. T. , Morrison, R. B. , Gorcyca, D. , & Chladek, D. (1992). Characterization of swine infertility and respiratory syndrome (SIRS) virus (isolate ATCC VR‐2332). Journal of Veterinary Diagnostic Investment, 4(2), 127–133. 10.1177/104063879200400202 1616976

[vms3699-bib-0002] Bi, C. , Shao, Z. , Zhang, Y. , Hu, L. , Li, J. , Huang, L. , & Weng, C. (2017). Identification of a linear B‐cell epitope on non‐structural protein 12 of porcine reproductive and respiratory syndrome virus, using a monoclonal antibody. Archives of Virology, 162(8), 2239–2246. 10.1007/s00705-017-3355-8 28365807

[vms3699-bib-0003] Brown, E. , Lawson, S. , Welbon, C. , Gnanandarajah, J. , Li, J. , Murtaugh, M. P. , Nelson, E. A. , Molina, R. M. , Zimmerman, J. J. , Rowland, R. R. , & Fang, Y. (2009). Antibody response to porcine reproductive and respiratory syndrome virus (PRRSV) nonstructural proteins and implications for diagnostic detection and differentiation of PRRSV types I and II. Clinical and Vaccine Immunology: CVI, 16, 628–635. 10.1128/CVI.00483-08 19261778 PMC2681581

[vms3699-bib-0004] Cancel‐Tirado, S. M. , Evans, R. B. , & Yoon, K. J. (2004). Monoclonal antibody analysis of porcine reproductive and respiratory syndrome virus epitopes associated with antibody‐dependent enhancement and neutralization of virus infection. Veterinary Immunology and Immunopathology, 102, 249–262. 10.1016/j.vetimm.2004.09.017 15507309 PMC7173136

[vms3699-bib-0005] Chand, R. J. , Trible, B. R. , & Rowland, R. R. R. (2012). Pathogenesis of porcine reproductive and respiratory syndrome virus. Current Opinion in Virology, 2(3), 256–263. 10.1016/j.coviro.2012.02.002 22709514

[vms3699-bib-0006] Darwich, L. , Díaz, I. , & Mateu, E. (2010). Certainties, doubts and hypotheses in porcine reproductive and respiratory syndrome virus immunobiology. Virus Research, 154, 123–132. 10.1016/j.virusres.2010.07.017 20659507

[vms3699-bib-0008] Díaz, I. , Pujols, J. , Ganges, L. , Gimeno, M. , Darwich, L. , Domingo, M. , & Mateu, E. (2009). In silico prediction and ex vivo evaluation of potential T‐cell epitopes in glycoproteins 4 and 5 and nucleocapsid protein of genotype‐I (European) of porcine reproductive and respiratory syndrome virus. Vaccine 27, 5603–5611. 10.1016/j.vaccine.2009.07.029 19646408

[vms3699-bib-0009] Flores‐Mendoza, L. , & Hernández, J. (2010). Vacunas contra el virus del síndrome reproductivo y respiratorio porcino (PRRSV): Escribiendo una historia. Veterinaria México 41, 139–159. http://www.scielo.org.mx/scielo.php?script=sci_arttext&pid=S0301-50922010000200007

[vms3699-bib-0010] García‐Plata, M. (2016). Desarrollo de una prueba de diagnóstico serológico (ELISA) para la detección de anticuerpos específicos en contra del virus del síndrome reproductivo y respiratorio porcino (PRRS) [Master´s Thesis], Maestría en Ciencias de la Producción y Salud Animal. Universidad Nacional Autónoma de México, México. http://132.248.9.195/ptd2016/febrero/0741360/Index.html

[vms3699-bib-0011] Gonin, P. , Pirzadeh, B. , Gagnon, C. A. , & Dea, S. (1999). Seroneutralization of porcine reproductive and respiratory syndrome virus correlates with antibody response to the GP5 major envelope glycoprotein. Journal of Veterinary Diagnostic Investigation, 11(1), 20–26. 10.1177/104063879901100103 9925207

[vms3699-bib-0012] Goyal, S. M. (1993). Porcine reproductive and respiratory syndrome. Journal of Veterinary Diagnostic Investigation, 5, 656–664. 8286480 10.1177/104063879300500435

[vms3699-bib-0013] Holtkamp, D. J. , Kliebenstein, J. B. , Neumann, E. J. , Zimmerman, J. J. , Rotto, H. F. , Yoder, T. K. , Wang, C. , Yeske, P. E. , Mowrer, C. L. , & Haley, C. A. (2013). Assessment of the economic impact of porcine reproductive and respiratory syndrome virus on United States pork producers. Journal of Swine Health and Production, 21(2), 72–84. https://www.aasv.org/shap/issues/v21n2/v21n2p72.pdf

[vms3699-bib-0014] Jiang, N. , Jin, H. , Li, Y. , Ge, X. , Han, J. , Guo, X. , Zhou, L. , & Yang, H. (2017). Identification of a novel linear B‐cell epitope in nonstructural protein 11 of porcine reproductive and respiratory syndrome virus that are conserved in both genotypes. PLoS One, 12(11), e0188946. 10.1371/journal.pone.0188946 29186182 PMC5706702

[vms3699-bib-0015] Johnson, C. R. , Yu, W. , & Murtaugh, M. P. (2007). Cross‐reactive antibody responses to nsp1 and nsp2 of Porcine reproductive and respiratory syndrome virus. Journal of General Virology, 88, 1184–1195. 10.1099/vir.0.82587-0 17374762

[vms3699-bib-0017] King, A. M. Q. , Lefkowitz, E. J. , Mushegian, A. R. , Adams, M. J. , Dutilh, B. E. , Gorbalenya, A. E. , Harrach, B. , Harrison, R. L. , Junglen, S. , Knowles, N. J. , Kropinski, A. M. , Krupovic, M. , Kuhn, J. H. , Nibert, M. L. , Rubino, L. , Sabanadzovic, S. , Sanfaçon, H. , Siddell, S. G. , Simmonds, P. , … Davison, A. J. (2018). Changes to taxonomy and the International Code of Virus Classification and Nomenclature ratified by the International Committee on Taxonomy of Viruses (2018). Archives of Virology, 163(9), 2601–2631. 10.1007/s00705-018-3847-1 29754305

[vms3699-bib-0018] Leng, C. , Zhang, W. , Zhang, H. , Kan, Y. , Yao, L. , Zhai, H. , Li, M. , Li, Z. , Liu, C. , An, T. , Peng, J. , Wang, Q. , Leng, Y. , Cai, X. , Tian, Z. , & Tong, G. (2017). ORF1a of highly pathogenic PRRS attenuated vaccine virus plays a key role in neutralizing antibody induction in piglets and virus neutralization in vitro. Virology journal, 14, 159. 10.1186/s12985-017-0825-2 28830563 PMC5568364

[vms3699-bib-0019] Loemba, H. D. , Mounir, S. , Mardassi, H. , Archambault, D. , & Dea, S. (1996). Kinetics of humoral immune response to the major structural proteins of the porcine reproductive and respiratory syndrome virus. Archives of Virology, 141(3‐4), 751–761. 10.1007/BF01718333 8645111 PMC7086943

[vms3699-bib-0020] Lopez, O. J. , & Osorio, F. A. (2004). Role of neutralizing antibodies in PRRSV protective immunity. Veterinary Immunology and Immunopathology, 102(3), 155–163. 10.1016/j.vetimm.2004.09.005 15507302

[vms3699-bib-0021] Lunney, J. K. , Benfield, D. A. , & Rowland, R. R. (2010). Porcine reproductive and respiratory syndrome virus: An update on an emerging and re‐emerging viral disease of swine. Virus Research, 154(1‐2), 1–6. 10.1016/j.virusres.2010.10.009 20951175 PMC7172856

[vms3699-bib-0022] Montaner‐Tarbes, S. , del Portillo, H. A. , Montoya, M. , Fraile, L. , & (2019). Key gaps in the knowledge of the porcine respiratory reproductive syndrome virus (PRRSV). Frontiers Veterinaria Science, 6, 38. 10.3389/fvets.2019.00038 PMC6391865 30842948

[vms3699-bib-0023] Nan, Y. , Wu, C. , Gu, G. , Sun, W. , Zhang, Y. J. , & Zhou, E. M. (2017). Improved vaccine against PRRSV: Current Progress and future perspective. Frontiers in Microbiology, 8, 1635. 10.3389/fmicb.2017.01635 28894443 PMC5581347

[vms3699-bib-0024] Neumann, E. J. , Kliebenstein, J. B. , Johnson, C. D. , Mabry, J. W. , Bush, E. J. , Seitzinger, A. H. , Green, A. L. , & Zimmerman, J. J. (2005). Assessment of the economic impact of porcine reproductive and respiratory syndrome on swine production in the United States. Journal of the American Veterinary Medical Association, 227, 385–392. 16121604 10.2460/javma.2005.227.385

[vms3699-bib-0025] Popescu, L. N. , Trible, B. R. , Chen, N. , & Rowland, R. R. R. (2017). GP5 of porcine reproductive and respiratory syndrome virus (PRRSV) as a target for homologous and broadly neutralizing antibodies. Veterinary Microbiology, 209, 90–96. 10.1016/j.vetmic.2017.04.016 28528961

[vms3699-bib-0026] Rascón‐Castelo, E. , Burgara‐Estrella, A. , Mateu, E. , & Hernández, J. (2015). Immunological features of the non‐structural proteins of porcine reproductive and respiratory syndrome virus. Viruses, 7, 873–886. 10.3390/v7030873 25719944 PMC4379552

[vms3699-bib-0027] Renukaradhya, G. J. , Meng, X.‐J. , Calvert, J. G. , Roof, M. , & Lager, K. M. (2015). Inactivated and subunit vaccines against porcine reproductive and respiratory syndrome: Current status and future direction. Vaccine, 33, 3065–3072. 10.1016/j.vaccine.2015.04.102 25980425

[vms3699-bib-0028] Rossow, K. D. (1998). Porcine Reproductive and Respiratory Syndrome. Veterinary Pathology, 35(1), 1–20. 10.1177/030098589803500101 9545131

[vms3699-bib-0016] Su, J. , Zhou, L. , He, B. , Zhang, X. , Ge, X. , Han, J. ,, Guo, X. & Yang, H. (2019). Nsp2 and GP5‐M of Porcine Reproductive and Respiratory Syndrome Virus Contribute to Targets for Neutralizing Antibodies. Virologica Sinica, 34(6), 631–640. 10.1007/s12250-019-00149-6 31347089 PMC6889258

[vms3699-bib-0029] Vashisht, K. , Goldberg, T. L. , Husmann, R. J. , Schnitzlein, W. , & Zuckermann, F. A. (2008). Identification of immunodominant T‐cell epitopes present in glycoprotein 5 of the North American genotype of porcine reproductive and respiratory syndrome virus. Vaccine, 26, 4747–4753. 10.1016/j.vaccine.2008.06.047 18590788

[vms3699-bib-0030] Welch, S. K. W. , & Calvert, J. G. (2010). A brief review of CD163 and its role in PRRSV infection. Virus Research, 154(1‐2), 98–103. 10.1016/j.virusres.2010.07.018 20655964

[vms3699-bib-0031] Wootton, S. K. , Nelson, E. a , & Yoo, D. (1998). Antigenic structure of the nucleocapsid protein of porcine reproductive and respiratory syndrome virus. Clinical and Diagnostic Laboratory Immunology, 5, 773–779. 9801333 10.1128/cdli.5.6.773-779.1998 PMC96200

[vms3699-bib-0032] Yoo, D. , Song, C. , Sun, Y. , Du, Y. , Kim, O. , & Liu, H. C. (2010). Modulation of host cell responses and evasion strategies for porcine reproductive and respiratory syndrome virus. Virus Research, 154(1‐2), 48–60. 10.1016/j.virusres.2010.07.019 20655963 PMC7114477

[vms3699-bib-0033] Yoon, I. J. , Joo, H. S. , Goyal, S. M. , & Molitor, T. W. (1994). A modified serum neutralization test for the detection of antibody to porcine reproductive and respiratory syndrome virus in swine sera. Journal of Veterinary Diagnostic Investigation, 6(3), 289–292. 10.1177/104063879400600326 7948196

